# Bioengineering of a human whole tooth: progress and challenge

**DOI:** 10.1186/2045-9769-3-8

**Published:** 2014-04-30

**Authors:** Yanding Zhang, YiPing Chen

**Affiliations:** 3 Fujian Key Laboratory of Developmental and Neuro Biology, College of Life Sciences, Fujian Normal University, Fuzhou, Fujian Province P.R. China; 4 Department of Cell and Molecular Biology, Tulane University, New Orleans, LA 70118 USA

## Abstract

A major challenge in stem cell-based bioengineering of an implantable human tooth is to identify appropriate sources of postnatal stem cells that are odontogenic competent as the epithelial component due to the lack of enamel epithelial cells in adult teeth. In a recent issue (2013, 2:6) of *Cell Regeneration*, Cai and colleagues reported that epithelial sheets derived from human induced pluripotent stem cells (iPSCs) can functionally substitute for tooth germ epithelium to regenerate tooth-like structures, providing an appealing stem cell source for future human tooth regeneration.

Stem cell-based tissue engineering is a promising approach to replace or repair lost or damaged tissues or even organs in humans. Two major types of stem cells currently used in tissue engineering research and clinical application are embryonic stem cells (ESCs) and adult stem cells. However, ethical concerns in using ESCs and difficulties in isolation, expansion, and differentiation of adult stem cells limit their clinical application. The advent of iPSCs has provided a promising alternative source of stem cells for tissue bioengineering [1].

Whole tooth bioengineering has given hope to dental replacement and regenerative therapy [2, 3]. In the last decade, techniques have been established to bioengineer a whole tooth crown from embryonic tooth germ cells in several animal models including mouse, rat, pig, and dog [4–11]. Remarkably, it was shown that re-aggregates of mouse embryonic tooth germ cells can develop into fully functional tooth replacement with revascularization and root formation in the lost tooth site of adult mice [12–14], demonstrating the feasibility of implanting a bioengineered tooth germ in adults for functional tooth replacement.

Tooth development relies on reciprocal tissue interactions between ectoderm-derived dental epithelium and cranial neural crest-derived mesenchyme [15]. In mice, the dental epithelium prior to embryonic day 12 (E12) possesses odontogenic potential (tooth-inducing capability) and is capable of inducing tooth formation when confronted with mesenchymal tissue of non-dental origin [16]. After E12, this odontogenic potential shifts to the dental mesenchyme that becomes capable of inducing tooth formation when recombined with non-dental epithelium. Thus, in vitro generation of a bioengineered whole tooth, which should follow the principles of tooth development, will also require epithelial and mesenchymal cells with either of them possessing the tooth-inducing capability. From the point view of clinical therapy, it is ideal to use patients’ own cells to generate bioengineered replacement teeth. At least five types of human postnatal mesenchymal stem cells of dental origin have been isolated thus far [17], including dental pulp stem cells (DPSCs), stem cells from exfoliated deciduous teeth (SHED), periodontal ligament stem cells (PDLSCs), dental follicle progenitor cells (DFPCs), and stem cells from the apical papilla (SCAP). However, epithelial stem cells of dental origin do not exist, because ameloblasts are lost via apoptosis upon tooth eruption [18].

Attempts have been made to identify alternative sources of human postnatal stem cells as the epithelial component for human whole tooth regeneration. It has been demonstrated that keratinocytes isolated from human foreskin and gingival epithelial cells isolated from patients’ gingival tissue are able to differentiate into enamel-secreting ameloblasts when recombined with mouse embryonic molar mesenchyme that possesses odontogenic potential [19, 20]. However, in these studies, epithelial cells were harvested from different individuals. The age, genetic variation, health condition, and even sex of each patient may influence the ultimate outcomes. A consistent cell source as the epithelial substitute would avoid these variations. In a recent issue of *Cell Regeneration* (2013, **2**:6), Cai and colleagues reported their studies on ameloblastic differentiation capability of integration-free human urine-derived iPSCs (ifhU-iPSCs) [21]. They first differentiated ifhU-iPSCs into epithelial cells with differentiation medium, followed by tissue recombination of the induced epithelial sheets with E14.5 mouse molar mesenchyme. Intact tooth-like structure formed in the recombinants after 3-week in subrenal culture in nude mice. Histological and molecular marker analyses demonstrated differentiation of ameloblasts from ifhU-iPSC derived epithelial cells as well as enamel formation. Interestingly, Nano-indentation and Raman spectroscopy analyses further revealed that the enamel deposited by ifhU-iPSC derived epithelial cells had similar physical properties and about 1/3 of hardness as compared to that in normal adult human teeth, suggesting the formation of functional enamel. Considering prolonged normal tooth development and differentiation in humans (about 400 days from the initiation of tooth development to tooth eruption), the formation of such structurally comparable enamel in the bioengineered teeth within 3 weeks indicates an adoption of accelerated developmental and differentiation programs in the human epithelial cells in response to the induction by the mouse embryonic dental mesenchyme, suggesting potential clinical application of accelerated differentiated tissues/organs from human iPSCs. While iPSC-derived cells have been widely tested for their capability in directed differentiation of various cell types, this study by Cai and colleagues appears to be the first in whole organ bioengineering with human iPSCs, indicating a realistic cell source for future in vitro generation or assembly of implantable tooth germs in dental practice.

Certainly, identification of novel adult human stem cell sources is not yet the final solution for tooth regeneration. Based on the characteristic features of tooth development, to generate an implantable tooth germ in vitro, one of the cell sources, either epithelial or mesenchymal population, must acquire odontogenic potential to initiate regenerative process. In fact, despite the fact that human iPSC-derived epithelial cells [21] as well as human keratinocytes [20] and gingival epithelial cells [19] are able to differentiate into enamel-secreting ameloblasts in response to tooth-inducing signals from the mouse embryonic dental mesenchyme, none of these cell populations possess odontogenic potential. In addition, all the human postnatal mesenchymal stem cells of dental origin identified so far, while capable of differentiating into various types of dental tissues, do not have tooth-inducing capability. Thus conferring cells with odontogenic potential is a major challenge. It is well established that epithelial-mesenchymal interactions are mediated by growth factors, and many growth factors are repeatedly utilized at different stages of tooth development [15]. The odontogenic potential must consist of a unique combination of growth factors in different tissue layer at certain stages of tooth development. While currently unknown, it has become a central importance to reveal the basis of the odontogenic potential. Thanks to rapid progress in molecular studies of tooth development, the expression profiles of numerous growth factors at different stages of tooth development have been well documented in mice. While slight differences exist in term of gene expression profiles in the developing tooth between mouse and human [22, 23], the fact that both mouse and human embryonic dental mesenchyme can equally induce tooth formation when recombined with human epithelial cells [19–21, 24] suggests a similar constitution of tooth-inducing signals. It is thus conceivable that an adult cell population that is directed to odontogenic fate with an expression profile of signaling molecules similar to that in an embryonic dental tissue with tooth-inducing capability will act as a “tooth inducer”. Since the dental mesenchyme determines the tooth type and size, it will be preferable to use a mesenchymal cell population as the “tooth inducer”. As outlined in a simplified blueprint of stem cell-based whole tooth regeneration with a scaffold-free approach (Figure 1), it is envisioned that iPSC derived epithelial cells or other appropriate adult cell populations will be prepared as the epithelial component. On the other hand, iPSC derived mesenchymal cells or dental mesenchymal stem cells will be manipulated to acquire odontogenic potential via induction or reprogramming approaches. Recombinants of the epithelial and mesenchymal components will be allowed to develop to the late bud or the early cap stage in vitro prior to being subjected to implantation into an extraction socket in a patient’s jaw. Because of the prolonged human tooth development and differentiation, it may be necessary to accelerate the development of the grafted tooth germ by manipulating gene expression via local application of small inhibitory molecules and growth factors. Ideally, the grafted tooth germs will be able to adjust to the local microenvironment including positional information to develop into a functional tooth. In conclusion, while many problems are waiting to be solved, fast progress in the molecular study of tooth biology and development thanks to new high-throughput technologies will no doubt facilitate realization of implantable bioengineered teeth.

**Figure 1:**
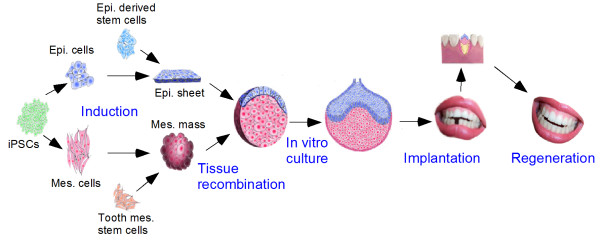
**A blueprint of stem cell-based tooth regeneration with a scaffold-free approach.** Schematic procedures of stem cell-based scaffold-free tooth regeneration in humans. The procedures include induction of iPSCs or epithelial derived stem cells into epithelial (epi.) sheets and induction of iPSCs or dental mesenchymal (mes.) stem cells into mesenchymal masses with odontogenic potential, tissue recombination, in vitro organ culture of the recombinants to the late bud or early cap stage, implantation of bioengineered tooth germs into the lost tooth sites of patients, and regeneration of functional replacement teeth.
